# A novel receptor-binding domain (RBD)-based mRNA vaccine against SARS-CoV-2

**DOI:** 10.1038/s41422-020-0387-5

**Published:** 2020-08-05

**Authors:** Wanbo Tai, Xiujuan Zhang, Aleksandra Drelich, Juan Shi, Jason C. Hsu, Larry Luchsinger, Christopher D. Hillyer, Chien-Te K. Tseng, Shibo Jiang, Lanying Du

**Affiliations:** 1 Lindsley F. Kimball Research Institute, New York Blood Center, New York, NY 10065 USA; 2 Department of Microbiology and Immunology, University of Texas Medical Branch, Galveston, TX 77555 USA

**Keywords:** Molecular biology, Immunology

Dear Editor,

The pandemic of coronavirus disease 2019 (COVID-19) caused by severe acute respiratory syndrome coronavirus 2 (SARS-CoV-2) highlights the need to develop effective and safe vaccines. Similar to SARS-CoV, SARS-CoV-2 recognizes angiotensin-converting enzyme 2 (ACE2) as receptor for host cell entry.^1,2^ SARS-CoV-2 spike (S) protein consists of S1, including receptor-binding domain (RBD), and S2 subunits.^3,4^ We previously demonstrated that RBDs of SARS-CoV and MERS-CoV serve as important targets for the development of effective vaccines.^5,6^

To identify an mRNA candidate vaccine, we initially designed two mRNA constructs expressing S1 and RBD, respectively, of SARS-CoV-2 S protein (Fig. 1a). Both culture supernatants and lysates of cells transfected with S1 or RBD mRNA reacted strongly with a SARS-CoV-2 RBD-specific antibody (Supplementary information, Fig. S1a), demonstrating expression of the target proteins.

**Fig. 1:**
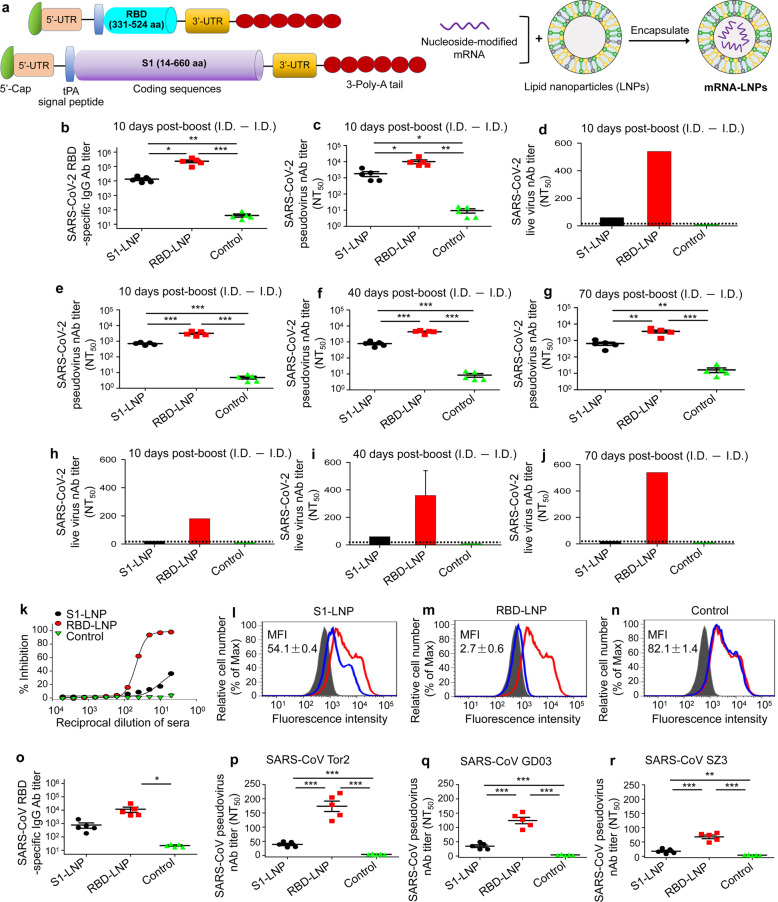
Design and evaluation of SARS-CoV-2 S1 and RBD mRNA vaccines. **a** Schematic diagram of SARS-CoV-2 S1 and RBD mRNA construction. The synthesized nucleoside-modified S1 and RBD mRNAs were encapsulated with LNPs to form mRNA-LNPs. **b**–**j** IgG and neutralizing antibodies induced in immunized BALB/c mice at different immunogen doses via intradermal (I.D.) prime and boost at 4 weeks. Sera at 10 days post-2nd immunization with SARS-CoV-2 S1 or RBD mRNA-LNP (e.g., S1-LNP or RBD-LNP) (30 μg/mouse), or empty LNP (control), were detected for SARS-CoV-2 RBD-specific IgG antibodies by ELISA (**b**) or neutralizing antibodies against pseudotyped (**c**) and live (**d**) SARS-CoV-2 infection. Sera at 10, 40, and 70 days post-2nd immunization with above mRNA-LNPs (10 μg/mouse) or control were detected for neutralizing antibodies against pseudotyped (**e**–**g**) and live (**h**–**j**) SARS-CoV-2 infection. The ELISA plates were coated with SARS-CoV-2 RBD-Fc protein (1 µg/ml), and IgG antibody (Ab) titer was calculated. Overall, 50% neutralizing antibody titer (nAb NT_50_) was calculated against SARS-CoV-2 pseudovirus infection in hACE2/293T cells, or against live SARS-CoV-2 infection by a cytopathic effect (CPE)-based microneutralization assay in Vero E6 cells. The dotted lines indicate detection limit. **k** Dose-dependent inhibition of sera of mice receiving a vaccine (30 μg/mouse) on SARS-CoV-2 RBD-hACE2 receptor binding in hACE2/293T cells by flow cytometry analysis. Percent (%) inhibition was calculated based on relative fluorescence intensity with or without respective serum at indicated dilutions. **l**–**n** Representative images of such inhibition by sera (1:5) of mice immunized with SARS-CoV-2 S1 mRNA-LNP (S1-LNP) (**l**), RBD mRNA-LNP (RBD-LNP) (**m**), or empty LNP control (**n**) are shown in blue lines with respective median fluorescence intensity (MFI) values. The binding between SARS-CoV-2 RBD-Fc protein (5 µg/mL) and hACE2 is shown in red lines. Gray shades indicate Fc-hACE2 binding. **o** Cross-reactivity of immunized mouse sera against SARS-CoV RBD by ELISA. SARS-CoV RBD-Fc protein-coated plates (1 µg/mL) were used to detect IgG Ab titer. **p**–**r** Cross nAb NT_50_ of above sera (twofold serial dilutions from 1:5) against infection of SARS-CoV pseudovirus expressing S protein of human SARS-CoV strains Tor2 (**p**) and GD03 (**q**), or palm civet SARS-CoV strain SZ3 (**r**) in hACE2/293T cells. Data (**b**, **c**, **e**–**g**, **k**–**r**) are presented as means ± SEM of mice (*n* = 5); data (**d**, **h**–**j**) are presented as means ± SEM of duplicate wells of pooled sera from five mice per group. Significant differences are shown as *P < 0.05; **P < 0.01; ***P < 0.001. Experiments were repeated twice with similar results.

To detect whether S1 and RBD mRNAs durably express antigens in multiple cell types, we constructed N-terminal mCherry-tagged SARS-CoV-2 S1 and RBD mRNAs, encapsulated them with lipid nanoparticles (LNPs) (Supplementary information, Fig. S1b), and tested mCherry expression. Relative to the control, both RBD- and S1-mCherry mRNAs showed robust protein expression in cells for at least 160 h, with higher expression of the RBD construct (Supplementary information, Fig. S2a). In addition, these mRNAs expressed proteins efficiently in a variety of human (A549, Hep-2, HEP-G2, Caco-2, HeLa, 293 T), monkey (Vero E6), and bat (Tb1-Lu) cell lines (Supplementary information, Fig. S2b). Particularly, the expression of RBD-mCherry protein was higher than that of S1-mCherry protein in all cell lines tested (Supplementary information, Fig. S2b). These data indicate long-term and broad expression of mRNA-encoding proteins, particularly RBD, in target cells.

We then characterized LNP-encapsulated S1 and RBD mRNAs for stability and subcellular localization. The mCherry-tagged S1 and RBD showed strong and stronger fluorescence intensity, respectively, irrespective of incubation temperature (4 or 25 °C) and culture time (0, 24, or 72 h) (Supplementary information, Fig. S3a). S1- and RBD-mCherry proteins were not colocalized with nuclei but associated with lysosomes (Supplementary information, Fig. S3b). These results suggest that LNP-encapsulated SARS-CoV-2 S1 and RBD mRNAs are stable at various temperatures and may be resistant to lysosomal degradation.

We next evaluated T follicular helper (Tfh), germinal center (GC) B, and plasma cell responses induced by SARS-CoV-2 S1 and RBD mRNA-LNPs in BALB/c mice. Mice were intradermally (I.D.) prime and boost immunized with each mRNA-LNP (30 μg/mouse) or empty LNP control, and draining lymph nodes or spleens were tested for Tfh, GC B, or plasma cells 10 days post-2nd immunization (Supplementary information, Fig. S4a). The percentages of Tfh cells (Supplementary information, Fig. S5a) and GC B cells (Supplementary information, Fig. S5b) were higher or significantly higher in the lymph nodes of RBD mRNA-LNP-immunized mice than in those of S1 mRNA-LNP-immunized mice, whereas only a background level of Tfh and GC B cells was shown in the LNP control-injected mice. Plasma cells were also significantly increased in splenocytes of the vaccinated mice, as compared to the control group (Supplementary information, Fig. S5c). These data demonstrate the recruitment of Tfh, GC B, and/or plasma cells *in vivo*, particularly after immunization with SARS-CoV-2 RBD mRNA-LNP vaccine.

We further evaluated humoral immune responses and neutralizing antibodies induced by S1 and RBD mRNA-LNPs. Mice were immunized with each mRNA-LNP at three different schedules (Supplementary information, Fig. S4a–c), and sera were collected for detection of IgG, subtype (IgG1 and IgG2a), and neutralizing antibodies. First, ELISA results revealed that S1 and RBD mRNA-LNPs (30 μg/mouse, I.D. prime and boost) induced RBD-specific IgG (Fig. 1b), IgG1 (Th2) (Supplementary information, Fig. S5d), and IgG2a (Th1) (Supplementary information, Fig. S5e) antibodies 10 days after boost immunization and that IgG antibody titer induced by RBD was significantly higher than that by S1 (Fig. 1b). Pseudovirus neutralization assay showed that S1 and RBD mRNA-LNPs elicited neutralizing antibodies against SARS-CoV-2 pseudovirus entry into human ACE2-expressing 293T (hACE2/293T) cells; particularly, RBD elicited significantly higher-titer neutralizing antibodies than S1 (Fig. 1c). Neutralizing antibodies, particularly those induced by RBD mRNA-LNP, also potently neutralized live SARS-CoV-2 infection (Fig. 1d). Next, both S1 and RBD mRNA-LNPs (10 μg, I.D. prime and boost) induced SARS-CoV-2 RBD-specific IgG (Supplementary information, Fig. S6a) and neutralizing antibodies against SARS-CoV-2 pseudovirus infection (Fig. 1e) 10 days after boost dose, and maintained at similarly high levels for 40 and 70 days post-boost immunization, while the titer of neutralizing antibodies elicited by RBD mRNA-LNP was always significantly higher than that by S1 mRNA-LNP (Fig. 1f, g; Supplementary information, S6b, c). Importantly, RBD mRNA-LNP induced antibody levels that potently neutralized live SARS-CoV-2 infection, reaching peak titer at 70 days post-2nd immunization and being significantly more potent than SARS-CoV-2 S1 mRNA-LNP-induced antibodies (Fig. 1h–j). Finally, RBD mRNA-LNP (10 μg, I.D. prime and intramuscular (I.M.) boost) also elicited significantly higher-titer RBD-specific IgG (Supplementary information, Fig. S6d) or neutralizing antibodies than S1 mRNA-LNP against SARS-CoV-2 pseudovirus (Supplementary information, Fig. S6g) and live SARS-CoV-2 (Supplementary information, Fig. S6j) infection 10 days after boost immunization, and such antibodies maintained at similar or even higher levels for at least 70 days post-boost dose (Supplementary information, Fig. S6e, f, h, i, k, l). In contrast, empty LNP control only elicited a background, or undetectable, level of antibodies incapable of neutralizing SARS-CoV-2 infection (Fig. 1b–j; Supplementary information, Fig. S6). These data suggest that RBD mRNA-LNP vaccine immunized at different immunogen doses and variant routes induced strong RBD-specific antibody responses and potent neutralizing antibodies against pseudotyped and live SARS-CoV-2 infection.

To substantiate antiviral activity, we found the binding of SARS-CoV-2 RBD to ACE2 receptor in hACE2/293T cells was inhibited by serum antibodies produced from RBD or S1 mRNA-LNP-vaccinated mice. Specifically, anti-RBD antibodies potently inhibited, in a dose-dependent manner, RBD-ACE2 receptor binding, which was much stronger than anti-S1 antibodies (Fig. 1k–m), while the control LNP-induced mouse sera did not inhibit RBD-ACE2 binding (Fig. 1k, n). These data suggest that RBD mRNA-LNP-induced antibodies can potently block binding between SARS-CoV-2 RBD and its ACE2 receptor.

Since SARS-CoV-2 RBD shares about 70% sequence identity with SARS-CoV RBD,^7^ we evaluated whether serum antibodies from SARS-CoV-2 mRNA-LNPs may cross-react with SARS-CoV RBD and neutralize SARS-CoV infection. ELISA results showed that the titer of IgG (Fig. 1o), IgG1 (Supplementary information, Fig. S5f), and IgG2a (Supplementary information, Fig. S5g) antibodies induced by SARS-CoV-2 RBD mRNA-LNP was higher, or significantly higher, than those by SARS-CoV-2 S1 mRNA-LNP in cross-reacting with SARS-CoV RBD and cross-neutralizing infection by three SARS-CoV pseudoviruses expressing S proteins of human strains Tor2 (Fig. 1p), GD03 (Fig. 1q), and palm civet strain SZ3 (Fig. 1r), respectively. These results suggest that SARS-CoV-2 RBD mRNA vaccine can elicit antibodies cross-reacting with SARS-CoV RBD and cross-neutralizing SARS-CoV infection.

We also investigated SARS-CoV-2 RBD-specific T cell responses induced by S1 and RBD mRNA-LNPs in immunized mice. Splenocytes collected 10 days post-2nd immunization were stimulated with SARS-CoV-2 RBD overlapping peptides (Supplementary information, Table S1), and detected for secretion of IFN-γ (Th1), TNF-α (Th1), and IL-4 (Th2) in CD45^+^CD4^+^ T cells, as well as IFN-γ, TNF-α, and IL-4 in CD45^+^CD8^+^ T cells by flow cytometry analysis. Compared with the LNP control, immunization with RBD mRNA-LNP could significantly increase the frequency of IFN-γ-, TNF-α- or IL-4-producing CD45^+^CD4^+^ (Supplementary information, Fig. S7a–c) or CD45^+^CD8^+^ (Supplementary information, Fig. S7d–f) T cells, respectively. However, S1 mRNA-LNP could only significantly increase the frequency of TNF-α-producing CD45^+^CD4^+^ (Supplementary information, Fig. S7b) and IFN-γ- or IL-4-producing CD45^+^-CD8^+^ (Supplementary information, Fig. S7d, f) T cells, respectively. Therefore, RBD mRNA vaccine can effectively elicit RBD-specific CD45^+^CD4^+^ (Th1) and CD45^+^CD8^+^ T cell responses.

As opposed to DNA, mRNA does not enter the nucleus and is not lysed by lysosomal enzymes (Supplementary information, Fig. S8),^8^ contributing to its high stability and translation efficiency. GC, where GC B cells interact with Tfh and B cells, is the major site for production of high-affinity antibodies.^9^ Here, we showed that RBD mRNA-LNP elicited strong Tfh and GC B cell responses and potent neutralizing antibodies able to inhibit the binding between SARS-CoV-2 RBD and ACE2 receptor (Supplementary information, Fig. S8), demonstrating its high potency against SARS-CoV-2 infection.

The repertoire of COVID-19 vaccines currently in clinical trials include mRNA, adenovirus, and DNA-based vaccines, most of which encode SARS-CoV-2 full-length S protein.^10–12^ It has been shown that adenovirus-based ChAdOx1 nCoV-19 vaccine elicits specific IgG antibody titer of 1:400–6400 and neutralizing antibody titer of 1:5–40, whereas the DNA vaccine induces a neutralizing antibody titer of 1:74–170, against live virus infection in immunized monkeys.^10,11^ In addition, neutralizing antibody titer against pseudotyped SARS-CoV-2 infection ranged from 1:89–1115 in mice immunized with a full-length S-based mRNA vaccine.^12^ Here we found that a SARS-CoV-2 RBD-based mRNA vaccine at 30 μg/mouse elicited SARS-CoV-2 RBD-specific IgG antibody titer (~1:230,000) and neutralizing antibody titer in mice against pseudotyped and live SARS-CoV-2 infection at ~1:10,000 and 1:540, respectively. Moreover, immunization with this vaccine at a lower immunogen dose (10 μg) via variant immunization routes (I.D. prime and I.D. or I.M. boost) also induced high-titer IgG antibodies with neutralizing activity against pseudotyped and live SARS-CoV-2 infection that persisted for at least 70 days during the detection period. Thus the IgG and neutralizing antibody titers induced by the RBD-based mRNA vaccine were higher than those reported, suggesting beneficial protection against SARS-CoV-2 challenge in vivo. Future studies warrant evaluation of protective efficacy using mRNA vaccine against other reported vaccines under development. Previous studies showed that SARS-CoV full-length S protein induced harmful immune responses with enhanced infection or liver damage after virus challenge, raising safety concerns.^6^ In contrast, RBD-based SARS-CoV and MERS-CoV vaccines had no evidence to cause harmful immune responses, including eosinophilic immune enhancement.^5,6^ Although no obvious adverse effects have been reported in currently developed COVID-19 vaccines, cautions need to be paid regarding their safety. More studies will be needed to investigate vaccine-associated immunopathology in addition to evaluate their protective efficacy.

Overall, this study identifies RBD as a key antigen to design effective vaccines against SARS-CoV-2, indicating great potential of RBD-based mRNA vaccine for mitigation of the COVID-19 pandemic and possible SARS-related epidemics in the future. The strategy of developing RBD-based mRNA COVID-19 vaccine, as described herein, can also be applied to develop vaccines against other emerging and reemerging coronavirus diseases in the future.

## Supplementary information

Supplementary Information
