# Supplementary material for: A novel receptor-binding domain (RBD)-based mRNA vaccine against SARS-CoV-2
Source: Cell Res. 2020 Aug 5;30(10):932–5. doi: 10.1038/s41422-020-0387-5 (PMC7403570; doi:10.1038/s41422-020-0387-5)
Supplement: Supplementary file 1 — Supplementary Information [file 41422_2020_387_MOESM1_ESM.pdf]

A novel receptor-binding domain (RBD)-based mRNA vaccine against SARS-CoV-2

Wanbo Tai[1#], Xiujuan Zhang[1#], Aleksandra Drelich[2], Juan Shi[1], Jason C Hsu[2], Larry Luchsinger[1], Christopher D Hillyer[1], Chien-Te K Tseng[2], Shibo Jiang[1*], Lanying Du[1*]

[1]Lindsley F. Kimball Research Institute, New York Blood Center, New York 10065, USA.

[2]Department of Microbiology and Immunology, University of Texas Medical Branch, Galveston, Texas 77555, USA.

[#]These authors contributed equally.

*Correspondence: sjiang@nybc.org (S.J.) or ldu@nybc.org (L.D.)

Supplementary Information

Materials and Methods

Design and synthesis of nucleoside-modified SARS-CoV-2 S1 and RBD mRNAs

SARS-CoV-2 S1 or RBD mRNAs with modified nucleosides were constructed and synthesized as follows. Briefly, genes encoding S1 (residues 14-660) and RBD (residues 331-524) of SARS-CoV-2 S protein were amplified using PCR and codon-optimized SARS-CoV-2 S plasmid (GenBank accession number QHR63250.1) as template. The amplified S1 or RBD (without mCherry tag) genes contained N-terminal T7 promotor, 5'-untranslated region (5'-UTR), tissue plasminogen activator (tPA) signal peptide and C-terminal 3'-UTR, and were inserted into pCAGGS-mCherry vector (Addgene, Watertown, MA, USA). To construct N-terminal mCherry-tagged S1 or RBD mRNAs, the above genes were amplified and fused to the C-terminal mCherry of this vector.

The mRNAs were synthesized, 5'-capped and tailed with 3'-Poly-A following manufacturer's instructions. Briefly, the above S1 or RBD genes were linearized using Bgl II enzyme and synthesized using the MEGAscript® T7 Kit (Thermo Fisher Scientific, Carlsbad, CA, USA), in the presence of CTP, ATP, GTP, and pseudouridine-5'-triphosphate (pseudouridine, Ψ) (MyChem, San Diego, CA, USA) (to replace uridine triphosphate UTP in order to increase stability and expression of target antigens). The synthesized and purified mRNAs were capped using the ScriptCap Cap 1 Capping System Kit (ScriptCap Capping Enzyme plus 2'-O-Methyltransferase) (CELLSCRIPT, Madison, WI, USA) to produce the Cap 1 structure, and tailed with Poly(A) Polymerase Tailing Kit (CELLSCRIPT, Madison, WI, USA) to obtain a Poly-A tail of about 150 base pair (bp). The mRNAs were stored at -80°C until use.

Preparation of SARS-CoV-2 S1 and RBD mRNA-LNPs

The above mRNAs were encapsulated with LNPs to further increase stability. mRNA-LNPs were prepared using GenVoy-ILM and NanoAssemblr Benchtop Instrument following manufacturer's instructions (Precision Nanosystems, Vancouver, BC). Briefly, GenVoy-ILM, which contains ionizable cationic lipid, helper lipids, and cholesterol, was dissolved in ethanol. The lipid mixture (ethanol phase) was encapsulated with PNI Formulation Buffer (aqueous phase) (Precision Nanosystems) containing SARS-CoV S1 or RBD mRNA (0.174 mg/ml) at 1:3 ratio (ethanol:aqueous, V/V), using NanoAssemblr Benchtop. The encapsulated mRNA-LNPs were diluted in PBS, filtered through a 0.22-mm filter, and concentrated using Amicon Ultra Centrifugal Filters (EMD Millipore, Billerica, MA, USA). The empty LNP control was prepared using PNI Formulation Buffer without mRNAs as aqueous phase. To assess consistency of physical characterization of LNPs among different batches, three batches of SARS-CoV-2 S1 mRNA-LNP, RBD mRNA-LNP, and empty LNP control were encapsulated by GenVoy-ILM and NanoAssemblr Benchtop Instrument as described above following manufacturer's instructions. The endotoxin level of each formulation was < 1 EU/ml. The particle size of LNPs was analyzed by Dynamic Light Scattering (Dynapro NanoStar), which was between 80-110 nm in diameter.

mRNA transfection and protein expression

mRNAs were transfected into 293T cells using TransIT-mRNA Kit following manufacturer's instructions (Mirus Bio, Madison, WI, USA). Briefly, SARS-CoV-2 S1 or RBD mRNA (1 μg) was mixed with TransIT-mRNA and boost reagents in Opti-Minimal Essential Medium (MEM). The mixture was added to cells containing complete Dulbecco's Modified Eagle's Medium (DMEM) and cultured at 37°C with 5% CO_2. 72 h after transfection, supernatants were collected, and cells were lysed in RIPA buffer (Sigma, St. Louis, MO, USA) for detection

of protein expression by Western blot. Samples were incubated with 4× Laemmli buffer (Bio-Rad, Hercules, California, USA), separated on a 10% polyacrylamide gel, and transferred to PVDF membrane, which was blocked with 5% fat-free milk in PBS containing 0.5% Tween-20 (PBST). S1 and RBD protein expression was detected by sequential incubation of the membrane with mouse sera (1:1,000) immunized with SARS-CoV-2 RBD-Fc protein and horseradish peroxidase (HRP)-conjugated goat anti-mouse IgG-Fab (1:5,000) (Sigma) for 1 h at room temperature. The signals were detected using ECL Western blot substrate reagents and Amersham Hyperfilm (GE Healthcare).

Flow cytometry

Flow cytometry was used to detect the expression of LNP-encapsulated SARS-CoV-2 S1 or RBD mRNA in different cells.[1,2] Briefly, human cell lines, including A549 (human lung), Hep-2 (human respiratory tract), HEP-G2 (human liver), Caco-2 (human intestinal tract), HeLa (human genitourinary tract), and 293T (human kidney), African green monkey kidney cells (Vero E6), and bat lung cells (Tb1-Lu) were pre-plated into 24-well culture plates (2×10^5/well) containing complete DMEM 24 h before experiments. The cells were then incubated with mCherry-tagged SARS-CoV-2 S1 or RBD mRNA-LNP (1 µg/ml) and cultured at 37°C. 48 h later or at indicated time points, the cells were collected for analysis of mCherry signal by flow cytometry (BD LSRFortessa 4 system).

Immunofluorescence staining

This was performed to detect subcellular localization of mRNA-encoding proteins as previously described with some modifications.[2,3] Briefly, mCherry-tagged SARS-CoV-2 S1 or RBD mRNA-LNP (1 µg/ml) was added to 293T cells (2×10^5/well) pre-plated 24 h before experiments; the cells were cultured at 37°C for 48 h and harvested for immunofluorescence

staining. The cells were then fixed and permed with FIX and PERM Cell Permeabilization Kit (Thermo Fisher Scientific), followed by incubation with FITC-labeled anti-human CD107a (LAMP-1, for lysosomes) antibody (1:100, BioLegend, San Diego, CA, USA) for 30 min at room temperature. After washing with PBS, the concentrated cell suspension was evenly distributed into slides, counter-stained with DAPI (4′,6-diamidino-2-phenylindole, 300 nM, Thermo Fisher Scientific) for nuclei for 5 min, and then mounted in VectaMount Permanent Mounting Medium (Vector Laboratories, Burlingame, CA, USA). The slides were imaged on a confocal microscope (Zeiss LSM 880). Images were prepared using ZEN software.

Thermal stability of mRNA-LNPs

The stability of LNP-encapsulated SARS-CoV-2 S1 or RBD mRNA was performed as described below. Briefly, mCherry-tagged SARS-CoV-2 S1 and RBD mRNA-LNPs were stored at 4°C and 25°C for 0, 24, and 72 h and then added to 293T cells at a concentration of 1 μg/ml. The cells were cultured at 37°C for 48 h and analyzed for mCherry signal by flow cytometry (BD LSRFortessa 4 system).

Ethics statement

Six-to-eight-week-old male and female BALB/c mice were used in the study. The animal protocols were approved by the Institutional Animal Care and Use Committee (IACUC) of New York Blood Center (permit number 194). All animal studies were carried out in strict accordance with the guidance and recommendations in the Guide for the Care and Use of Laboratory Animals (National Research Council Committee).

Mouse immunization and sample collection

Three immunization protocols were performed as previously described with some modifications.[1,4] First, mice were intradermally (I.D.) immunized with SARS-CoV-2 S1 or RBD mRNA-LNP (30 μg/100 μl/mouse), or empty LNP (control), and I.D. boosted at 4 weeks with the same immunogens (I.D. – I.D.). Ten days after the 2nd immunization, lymph nodes were collected to detect T follicular helper (Tfh) and germinal center (GC) B, sera were collected to detect antibody response and neutralizing antibodies, and spleens were collected to detect plasma cells and SARS-CoV-2-specific T cell responses, as described below. Second, mice were I.D. immunized with SARS-CoV-2 S1 or RBD mRNA-LNP (10 μg/100 μl/mouse), or empty LNP (control), and I.D. boosted at 4 weeks with the same immunogens (I.D. – I.D.). Sera were collected at 10, 40, and 70 days post-2nd immunization, and detected for antibody response and neutralizing antibodies. Third, mice were I.D. immunized with SARS-CoV-2 S1 or RBD mRNA-LNP (10 μg/100 μl/mouse), or empty LNP (control), and intramuscularly (I.M.) boosted at 4 weeks with the same immunogens (I.D. – I.M.). Sera were collected at 10, 40, and 70 days post-2nd immunization, and detected for antibody responses and neutralizing antibodies.

ELISA

ELISA was performed to detect SARS-CoV-2 or SARS-CoV RBD-specific antibodies in immunized mouse sera as previously described.[2] Briefly, ELISA plates were coated with SARS-CoV-2 or SARS-CoV RBD-Fc protein[2] (1 μg/ml) overnight at 4°C and blocked with 2% fat-free milk in PBST for 2 h at 37°C. After three washes with PBST, the plates were sequentially incubated with serially diluted mouse sera and HRP-conjugated anti-mouse IgG (1:5,000), IgG1 (1:5,000), or IgG2a (1:2,000) antibodies (Thermo Fisher Scientific) for 1 h at 37°C. The plates were sequentially incubated with substrate TMB (3,3',5,5'-tetramethylbenzidine) (Sigma) and then H_2SO_4 (1N) to stop the reaction. Absorbance at 450 nm was measured using an ELISA plate reader (Tecan).

Pseudovirus neutralization assay

mRNA-LNP vaccine-induced neutralizing antibodies against SARS-CoV-2 and SARS-CoV pseudovirus infection were detected using our established pseudovirus neutralization assay.[2] Briefly, 293T cells were co-transfected with a plasmid encoding S protein of SARS-CoV-2 (GenBank accession number QHR63250.1) or SARS-CoV Tor2 strain (GenBank accession number AY274119) and a plasmid encoding Env-defective, luciferase-expressing HIV-1 genome (pNL4-3.luc.RE). SARS-CoV GD03 (GD03T0013, GenBank accession number AY525636) and SARS-CoV SZ3 (GenBank accession number AY304486) pseudoviruses were prepared as described above except for using Tor2 S protein-encoding plasmid containing mutations for GD03 and SZ3 at their respective RBD regions.[5] Culture supernatants containing pseudoviruses were collected at 72 h after transfection, incubated with serially diluted mouse sera for 1 h at 37°C, added to 293T cells expressing SARS-CoV-2 or SARS-CoV receptor human angiotensin-converting enzyme 2 (ACE2) (hACE2/293T), and then cultured at 37°C. The cells were lysed using cell lysis buffer (Promega, Madison, WI, USA) 72 h post-culture and transferred into luminometer plates. Luciferase substrate (Promega) was added to the plates and measured for relative luciferase activity using the Infinite 200 PRO Luminometer (Tecan). Neutralizing activity of serum antibodies against SARS-CoV-2 and SARS-CoV pseudoviruses was calculated using the CalcuSyn computer progra[6,7] and expressed as 50% pseudovirus neutralizing antibody titer (NT_{50}).

SARS-CoV-2 microneutralization assay

mRNA-LNP vaccine-induced serum neutralizing antibodies against live SARS-CoV-2 infection were detected using a cytopathic effect (CPE)-based microneutralization assay as previously described with some modifications.[6,8] Briefly, Vero E6 cells (10^4/well) were pre-

plated in 96-well tissue culture plates and cultured at 37°C to form a monolayer. Serially 2 or 3-fold and duplicate dilutions of mouse sera (pooled from five mice in each group) were thoroughly mixed with ~120 median tissue culture infectious dose ($TCID_{50}$) of SARS-CoV-2 (US-WA-1 isolate), and incubated for 1 h at room temperature, followed by transfer of the serum-virus mixtures to the above cells and culture of the cells at 37°C for three days. Cells with or without virus were included as positive and negative controls, respectively. CPE in each well was observed under the microscopy, and recorded on day 3 post-infection. Neutralizing antibody titer was calculated and expressed as the highest dilutions of mouse sera capable of completely preventing virus-induced CPE in at least 50% of the wells (NT_{50}).

Inhibition of binding of SARS-CoV-2 RBD protein to hACE2 receptor

Flow cytometry was used to analyze the ability of immunized mouse sera to block the binding between SARS-CoV-2 RBD protein and cell-associated hACE2 receptor.[2] Briefly, hACE2/293T cells were incubated with SARS-CoV-2 RBD-Fc protein (5 µg/ml) in the presence or absence of serially diluted mouse sera for 30 min at room temperature. The cells were stained with FITC-labeled goat anti-human IgG-Fc antibody (1:500, Sigma) for 30 min at room temperature and measured for fluorescence by flow cytometry (BD LSRFortessa 4 system).

Isolation and analysis of lymph node cells

Lymph nodes collected at 10 days post-2nd immunization (I.D.) with SARS-CoV-2 mRNA-LNP or empty LNP were pooled for detection as described below.[9] Briefly, lymph nodes were homogenized into single cell suspensions in complete DMEM and filtered through a cell strainer (70 µm). The isolated cells were washed, resuspended in PBS containing 2% FBS, and stained with Fixable Viability Dye eFluor™ 780 (Thermo Fisher Scientific) for live and dead

cells. The cells were then stained with fluorescence-labeled antibody cocktails, including anti-mouse CD45-AF700, CD4-PE/Cy7, CD185 (CXCR5)-BV605, PD-1-BV421, B220-PerCP/Cy5.5, GL-7-APC (BioLegend), and CD95-BV510 (BD Biosciences, San Jose, CA, USA), and incubated in dark for 20 min at room temperature. The stained cells were washed, resuspended in Cell Staining Buffer (BioLegend), and analyzed for Tfh and GC B cells using flow cytometry (BD LSRFortessa 4 system). The data were analyzed using FlowJo software.

Plasma cell, surface and intracellular staining

Splenocytes collected at 10 days post-2nd immunization (I.D.) with SARS-CoV-2 mRNA-LNP or empty LNP were detected for plasma cell and RBD-specific T cell immune responses.[10,11] Splenocytes from homogenized spleens were resuspended in complete DMEM, treated with 1 × Red Blood Cell Lysis Buffer (BioLegend), washed with PBS, and resuspended in complete DMEM. To detect plasma cells, splenocytes (1×10^6) were stained with a cocktail of antibodies including anti-mouse CD45-AF700, CD27-BV421, B220-PerCP/Cy5.5, and CD138-PE (Biolegend) in Cell Staining Buffer (Biolegend). To detect RBD-specific T cell responses, splenocytes (1×10^6) were incubated with a mixture of overlapping SARS-CoV-2 RBD peptides (5 μg/mL) (Supplementary information, Table S1) and cultured at 37°C for 72 h. At 68 h post-stimulation, 1× Brefeldin A (BioLegend) was added to the cells. After stimulation, the cells were washed with PBS and stained with Fixable Viability Dye eFluor™ 780 (Thermo Fisher Scientific) for live and dead cells. The cells were stained for surface markers using anti-mouse CD45-AF700, CD4-FITC, and CD8-PerCP/Cy5.5 antibodies (BioLegend). After fixation and permeabilization, the cells were stained for intracellular cytokine markers using IFN-γ-PE, TNF-α-BV421, and IL-4-BV711 (BioLegend). The stained cells were measured using flow cytometry (BD LSRFortessa 4 system), and the data were analyzed using FlowJo software.

Statistical analyses

All values are presented as mean plus standard error of the mean (s.e.m). Statistical differences among SARS-CoV-2 S1 mRNA-LNP, RBD mRNA-LNP and control groups, as shown in Fig. 1 and Supplementary information, Fig. S5-7, were performed using Student's two-tailed *t*-test. * ($P < 0.05$), ** ($P < 0.01$), and *** ($P < 0.001$) represent significance and high significance among different groups. All statistical analyses were performed using GraphPad Prism 5 statistical software.

Supplementary References

1. Tai, W. et al. Recombinant receptor-binding domains of multiple Middle East respiratory syndrome coronaviruses (MERS-CoVs) induce cross-neutralizing antibodies against divergent human and camel MERS-CoVs and antibody escape mutants. *J. Virol.* **91**, (2017).
2. Tai, W. et al. Characterization of the receptor-binding domain (RBD) of 2019 novel coronavirus: implication for development of RBD protein as a viral attachment inhibitor and vaccine. *Cell. Mol. Immunol.* **17**, 613-620 (2020).
3. Tai, W. et al. Transfusion-transmitted Zika virus infection in pregnant mice leads to broad tissue tropism with severe placental damage and fetal demise. *Front. Microbiol.* **10**, 29 (2019).
4. Du, L. et al. Introduction of neutralizing immunogenicity index to the rational design of MERS coronavirus subunit vaccines. *Nat. Commun.* **7**, 13473 (2016).
5. He, Y. et al. Cross-neutralization of human and palm civet severe acute respiratory syndrome coronaviruses by antibodies targeting the receptor-binding domain of spike protein. *J. Immunol.* **176**, 6085-6092 (2006).
6. Du, L. et al. Recombinant receptor-binding domain of SARS-CoV spike protein expressed in mammalian, insect and E. coli cells elicits potent neutralizing antibody and protective immunity. *Virology* **393**, 144-150 (2009).
7. Tai, W. et al. Rational design of Zika virus subunit vaccine with enhanced efficacy. *J. Virol.* **93**, (2019).
8. Du, L. et al. Antigenicity and immunogenicity of SARS-CoV S protein receptor-binding domain stably expressed in CHO cells. *Biochem. Biophys. Res. Commun.* **384**, 486-490 (2009).
9. Wang, Z. et al. A novel rabies vaccine expressing CXCL13 enhances humoral immunity by recruiting both T follicular helper and germinal center B cells. *J. Virol.* **91**, (2017).

10. Ma, C. et al. Intranasal vaccination with recombinant receptor-binding domain of MERS-CoV spike protein induces much stronger local mucosal immune responses than subcutaneous immunization: Implication for designing novel mucosal MERS vaccines. *Vaccine* **32**, 2100-2108 (2014).
11. Wang, X. et al. Effects of adjuvants on the immunogenicity and efficacy of a Zika virus envelope domain III subunit vaccine. *Vaccines (Basel)* **7**, (2019).

Supplementary Figures

Supplementary information, Fig. S1

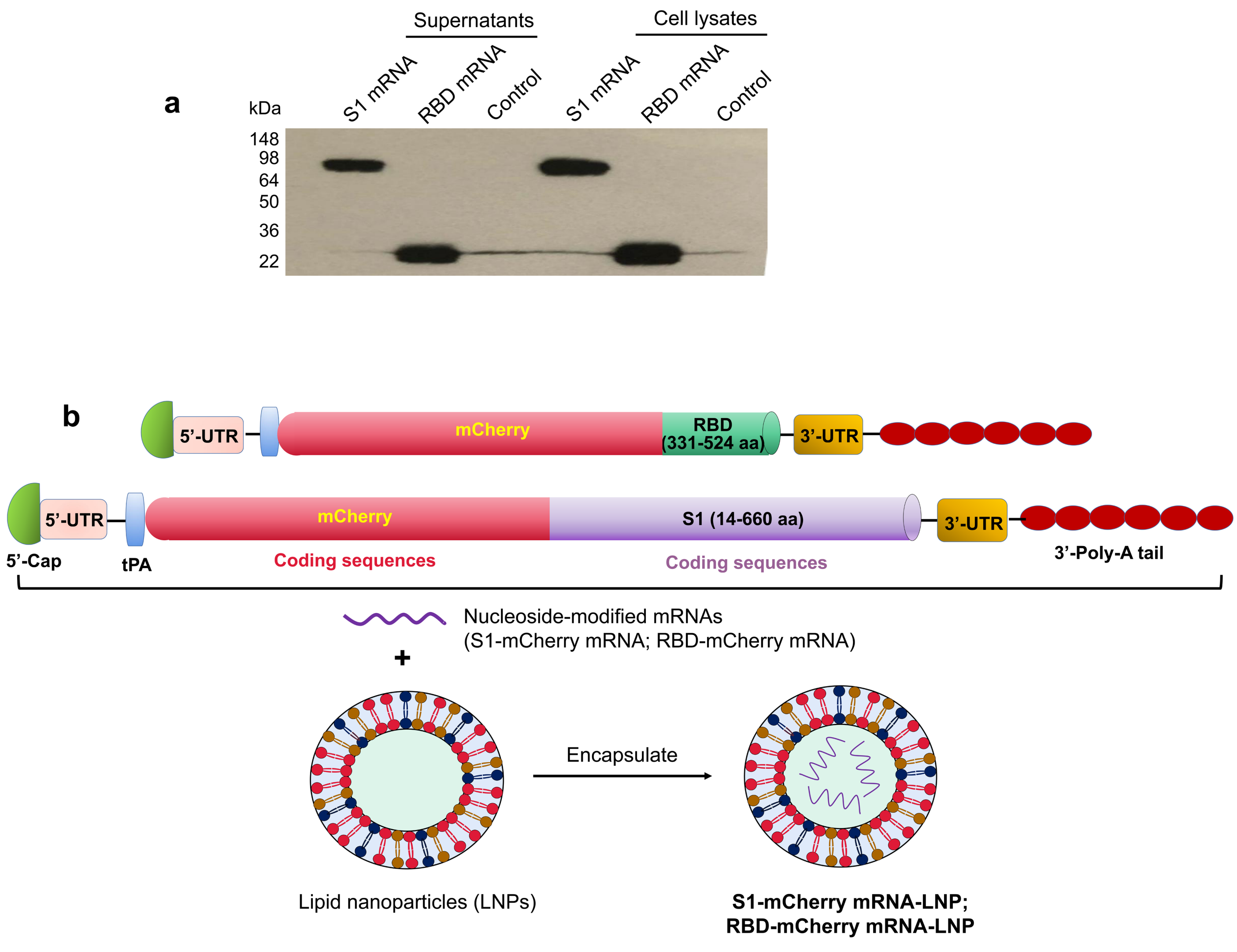

Supplementary information, Fig. S1 Expression of RBD protein encoded by nucleoside-modified SARS-CoV-2 S1 and RBD mRNAs and design of mCherry-tagged SARS-CoV-2 S1 and RBD mRNAs.

a Analysis of expression of SARS-CoV-2 S1 and RBD mRNA-encoding RBD protein by Western blot. The mRNAs were transfected into 293T cells, and cell lysates and supernatants were collected 48 h post-transfection to detect protein expression using sera (polyclonal antibody, 1:1,000 dilution) of mice immunized with SARS-CoV-2 RBD-Fc protein. Mock cells were used as negative control. Protein molecular weight marker (kDa) is shown on the left. **b** Design of nucleoside-modified SARS-CoV-2 S1 and RBD mRNAs fused with an N-terminal mCherry tag. Each mRNA consists of a 5'-Cap (with the Cap 1 structure), 5'-untranslated region (UTR), a tPA signal peptide with nucleoside-modified coding sequences (mCherry and S1 or RBD of SARS-CoV-2), 3'-UTR, and a 3'-Poly-A tail. The synthesized nucleoside-modified mRNAs (containing pseudouridine (Ψ) instead of uridine triphosphate (UTP)) were encapsulated with lipid nanoparticles (LNPs) to form mCherry-tagged SARS-CoV-2 S1 or RBD mRNA-LNPs.

Supplementary information, Fig. S2

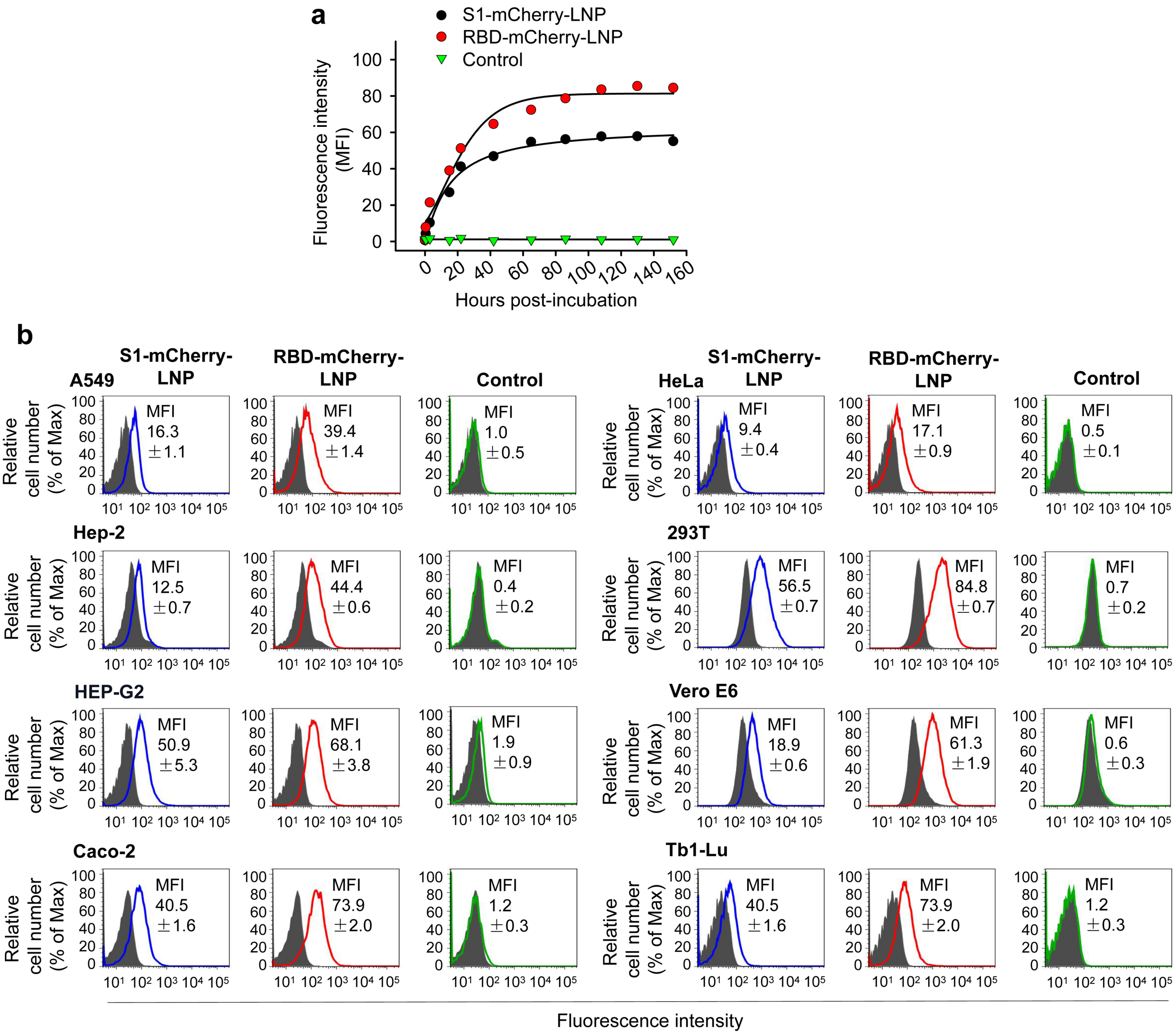

Supplementary information, Fig. S2 Expression of mCherry protein encoded by nucleoside-modified mCherry-tagged SARS-CoV-2 S1 and RBD mRNAs.

a Long-term expression of mCherry protein encoded by mCherry-tagged S1 and RBD mRNAs in 293T cells. The LNP-encapsulated mRNAs encoding SARS-CoV-2 S1 or RBD protein (S1-mCherry-LNP or RBD-mCherry-LNP) were incubated with 293T cells at 37°C, and the cells were then collected at different time post-incubation for analysis of mCherry signal by flow cytometry. **b** Broad-spectrum expression of mCherry protein encoded by mCherry-tagged S1 and RBD mRNAs in different human, monkey, and bat cells. The LNP-encapsulated S1 or RBD mRNA (S1-mCherry-LNP or RBD-mCherry-LNP) was incubated with each cell line at 37°C for 48 h and analyzed for mCherry signal by flow cytometry. Data in (**a**) to (**b**) are presented as median fluorescence intensity (MFI) ± standard error of the mean (s.e.m.) of triplicates (n = 3). Control, empty LNP. Experiments were repeated twice with similar results.

Supplementary information, Fig. S3

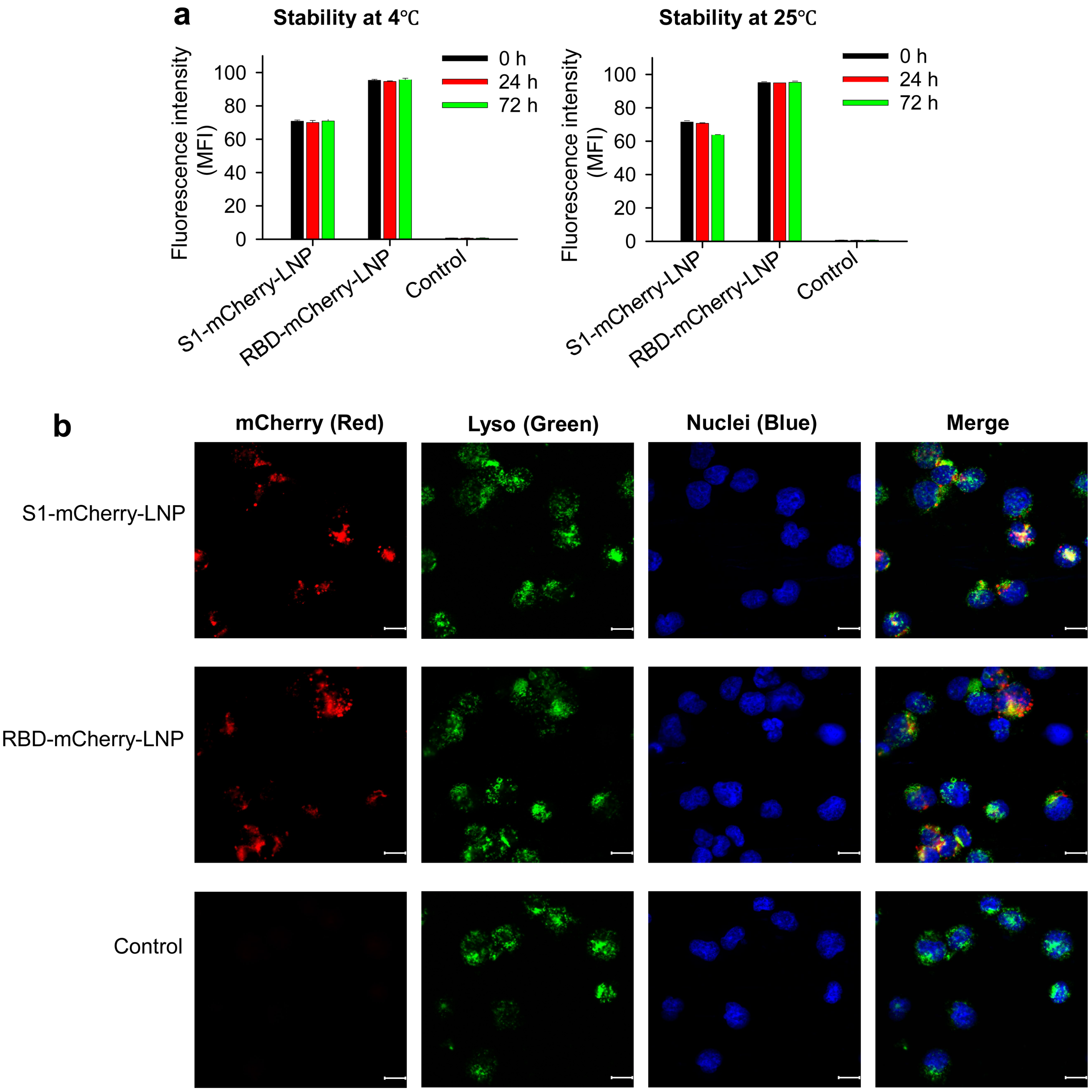

Supplementary information, Fig. S3 Characterization of SARS-CoV-2 S1 and RBD mRNA-LNPs.

a Stability of LNP-encapsulated, mCherry-tagged SARS-CoV-2 S1 and RBD mRNAs (S1-mCherry-LNP or RBD-mCherry-LNP). The mRNAs were stored at 4°C and 25°C, respectively, at the indicated time and then incubated with 293T cells at 37°C for 48 h, followed by analysis for mCherry signal by flow cytometry. Data are presented as mean MFI ± s.e.m. of triplicates (n = 3). **b** Detection of subcellular localization of mRNA-encoding protein. LNP-encapsulated, mCherry-tagged SARS-CoV-2 S1 and RBD mRNAs (S1-mCherry-LNP or RBD-mCherry-LNP) were incubated with 293T cells at 37°C for 48 h. Cell lysosomes (Lyso, green) and nuclei (blue) were stained, and subcellular localization of mRNA expression based on mCherry (red) signal was analyzed by immunofluorescence microscope. Representative images are shown. Scale bar, 10 μm. Control, empty LNP. Experiments were repeated twice with similar results.

Supplementary information, Fig. S4

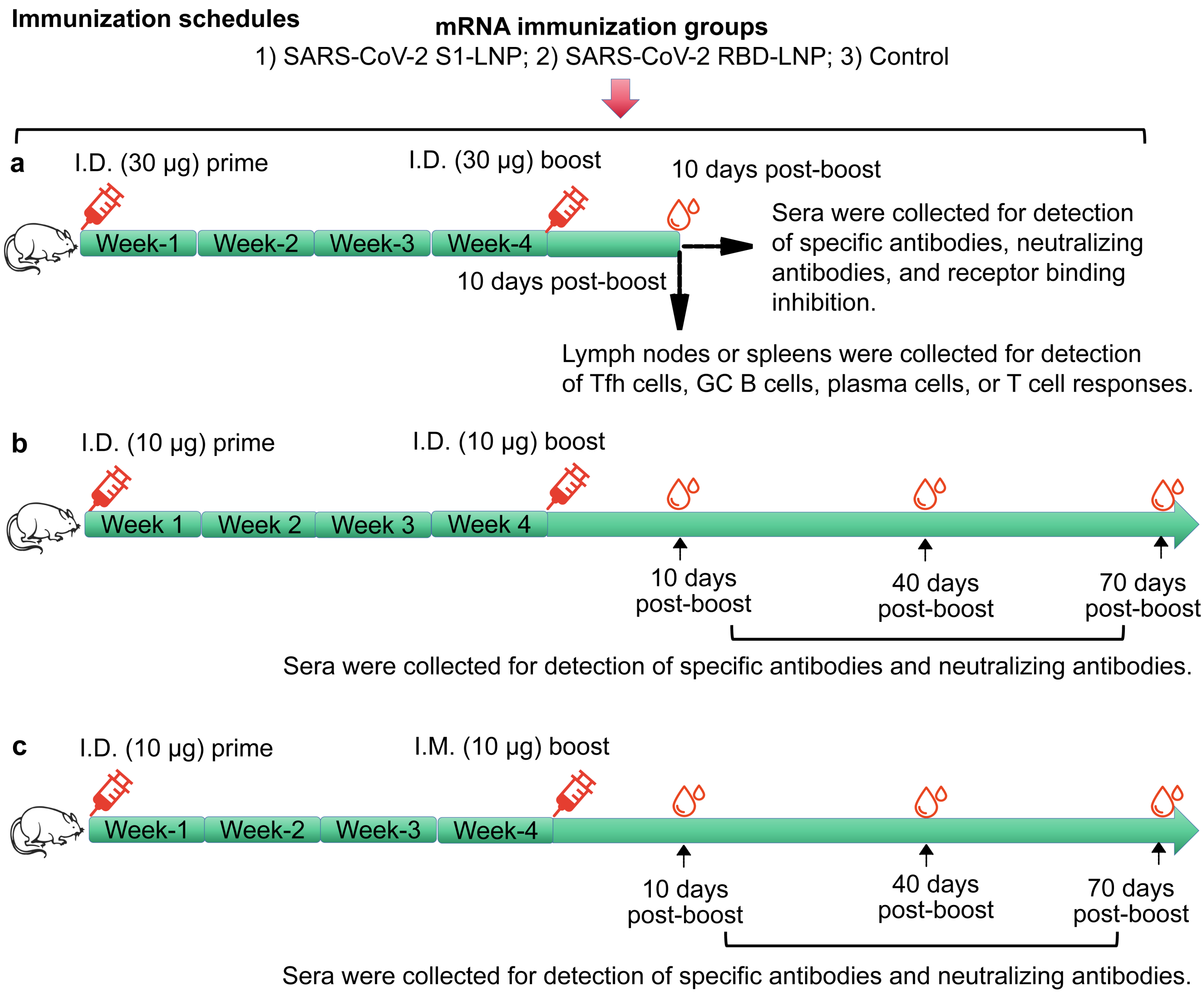

Supplementary information, Fig. S4 Immunization schedules of SARS-CoV-2 S1 and RBD mRNA-LNPs.

BALB/c mice at 6-8 weeks old were immunized with SARS-CoV-2 S1 or RBD mRNA-LNP (S1-LNP or RBD-LNP), or control (empty LNP) for three vaccination schedules. **a** Mice were I.D. primed and boosted with each mRNA-LNP (30 μg/mouse) or empty LNP control. Ten days post-2nd immunization, mouse lymph nodes or spleens were collected to detect Tfh cells, GC B cells, plasma cells, or T cell responses, and sera were collected to detect specific antibodies, neutralizing antibodies, and inhibition of receptor binding. **b** Mice were I.D. primed and boosted with each mRNA-LNP (10 μg/mouse) or empty LNP control, and sera were collected at 10, 40, and 70 days post-2nd immunization to detect specific antibody responses and neutralizing antibodies. **c** Mice were I.D. primed and I.M. boosted with each mRNA-LNP (10 μg/mouse) or empty LNP control, and sera were collected at 10, 40, and 70 days post-2nd immunization to detect specific antibody responses and neutralizing antibodies.

Supplementary information, Fig. S5

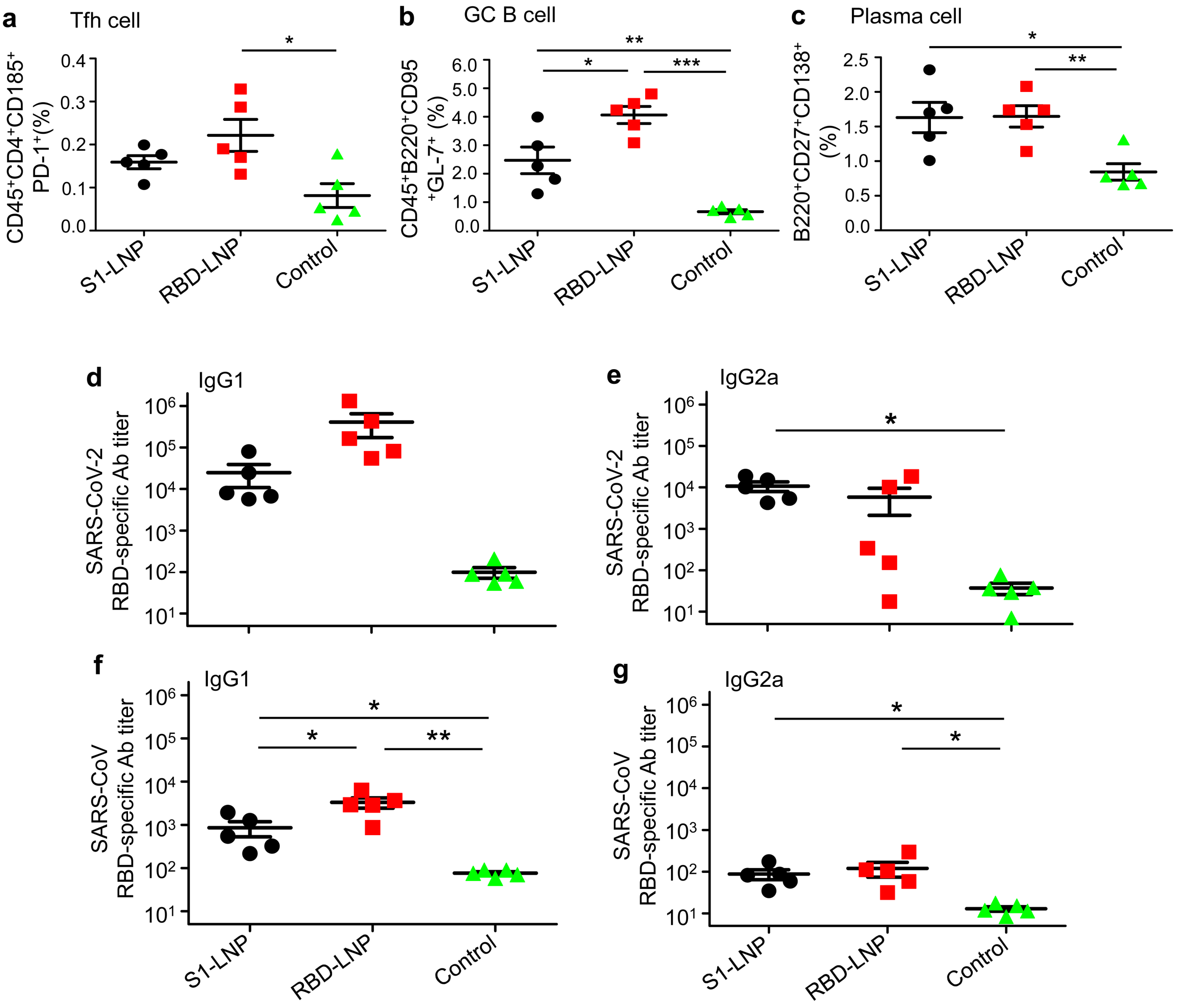

Supplementary information, Fig. S5 SARS-CoV-2 RBD mRNA-LNP elicited Tfh and GC B cell responses and subtype antibody responses specific to SARS-CoV-2 and SARS-CoV RBDs.

BALB/c mice were I.D. immunized with LNP-encapsulated SARS-CoV-2 S1 or RBD mRNA (S1-LNP or RBD-LNP) (30 μg/mouse), or empty LNP (control), I.D. boosted at 4 weeks, and lymph nodes, spleens, and sera were collected at 10 days post-2nd immunization. **a-c** Detection of Tfh cells (**a**), GC B cells (**b**), and plasma cells (**c**) in the immunized mouse lymph nodes (for Tfh and GC B cells) and spleens (for plasma cells). Frequencies of Tfh (CD45$^+$CD4$^+$CD185$^+$PD-1$^+$), GC B (CD45$^+$B220$^+$CD95$^+$GL-7$^+$), and plasma (B220$^+$CD27$^+$CD138$^+$) cells were analyzed by flow cytometry. Data in (**a**) to (**c**) are presented as mean ± s.e.m. of mice (n = 5). **d-g** Detection of SARS-CoV-2 RBD-specific IgG1 (**d**) and IgG2a (**e**), as well as SARS-CoV RBD-specific IgG1 (**f**) and IgG2a (**g**), antibodies in sera by ELISA. The plates were coated with SARS-CoV-2 RBD-Fc (for SARS-CoV-2) or SARS-CoV RBD-Fc (for SARS-CoV) protein (1 μg/ml), and IgG1 and IgG2a antibody (Ab) titers were calculated as the endpoint dilution that remained positively detectable. Data are presented as mean ± s.e.m. of mice (n = 5). Significant differences (*, $P < 0.05$; **, $P < 0.01$; ***, $P < 0.001$) among different groups are shown in related figures. Experiments were repeated twice with similar results.

Supplementary information, Fig. S6

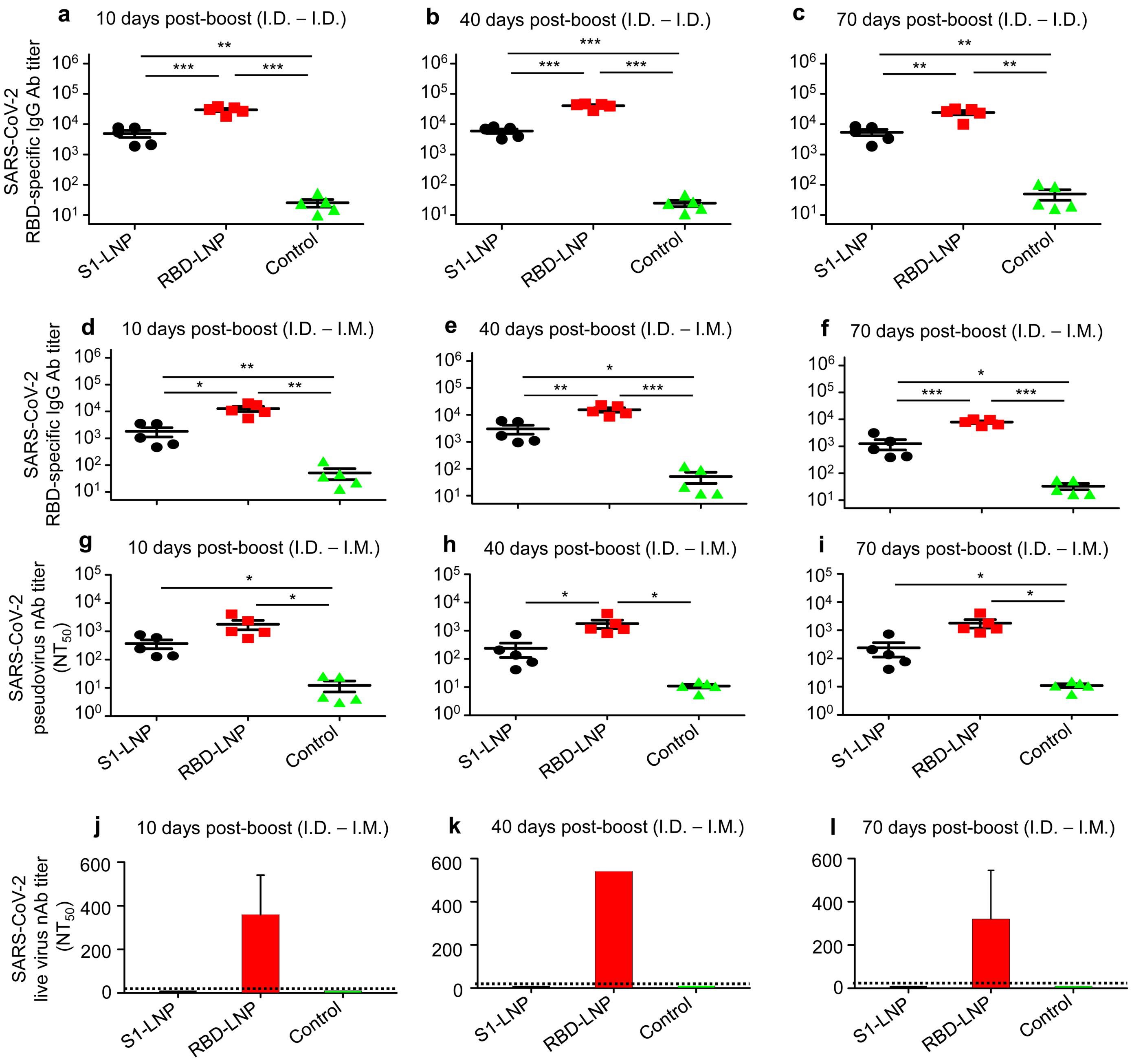

Supplementary information, Fig. S6 SARS-CoV-2 RBD mRNA-LNP at a low immunogen dose or different routes elicited potent antibodies with strong neutralizing activity against SARS-CoV-2 infection.

a-l BALB/c mice were I.D. immunized with SARS-CoV-2 S1 mRNA-LNP or RBD mRNA-LNP (S1-LNP or RBD-LNP) (10 μg/mouse), or empty LNP (control), and I.D. (**a-c**) or I.M. (**d-l**) boosted at 4 weeks. SARS-CoV-2 RBD-specific IgG antibodies in sera of mice (I.D. prime-I.D. boost) collected at 10 (**a**), 40 (**b**), and 70 (**c**) days post-2nd immunization were detected by ELISA. SARS-CoV-2 RBD-specific IgG antibodies (**d-f**) and neutralizing antibodies against SARS-CoV-2 pseudovirus (**g- i**) and live SARS-CoV-2 (**j-l**) infection in sera of mice (I.D. prime-I.M. boost) collected at 10, 40, and 70 days post-2nd immunization were detected by ELISA and neutralization assays, respectively. The ELISA plates were coated with SARS-CoV-2 RBD-Fc protein (1 μg/ml), and IgG antibody (Ab) titer was calculated as the endpoint dilution that remained positively detectable. The data are presented as mean ± s.e.m. of mice (n = 5). 50% pseudovirus neutralizing antibody titer (nAb NT_{50}) was calculated against SARS-CoV-2 pseudovirus infection in hACE2/293T cells, and the data are presented as mean ± s.e.m. of mice (n = 5). 50% live SARS-CoV-2 neutralizing antibody (nAb NT_{50}) titer was calculated against live SARS-CoV-2 infection in Vero E6 cells, and the data are presented as mean ± s.e.m. of duplicate wells of pooled sera from five mice in each group. The dotted lines indicate the detection limit. Significant differences (*, $P < 0.05$; **, $P < 0.01$; ***, $P < 0.001$) among different groups are shown in related figures. Experiments were repeated twice with similar results.

Supplementary information, Fig. S7

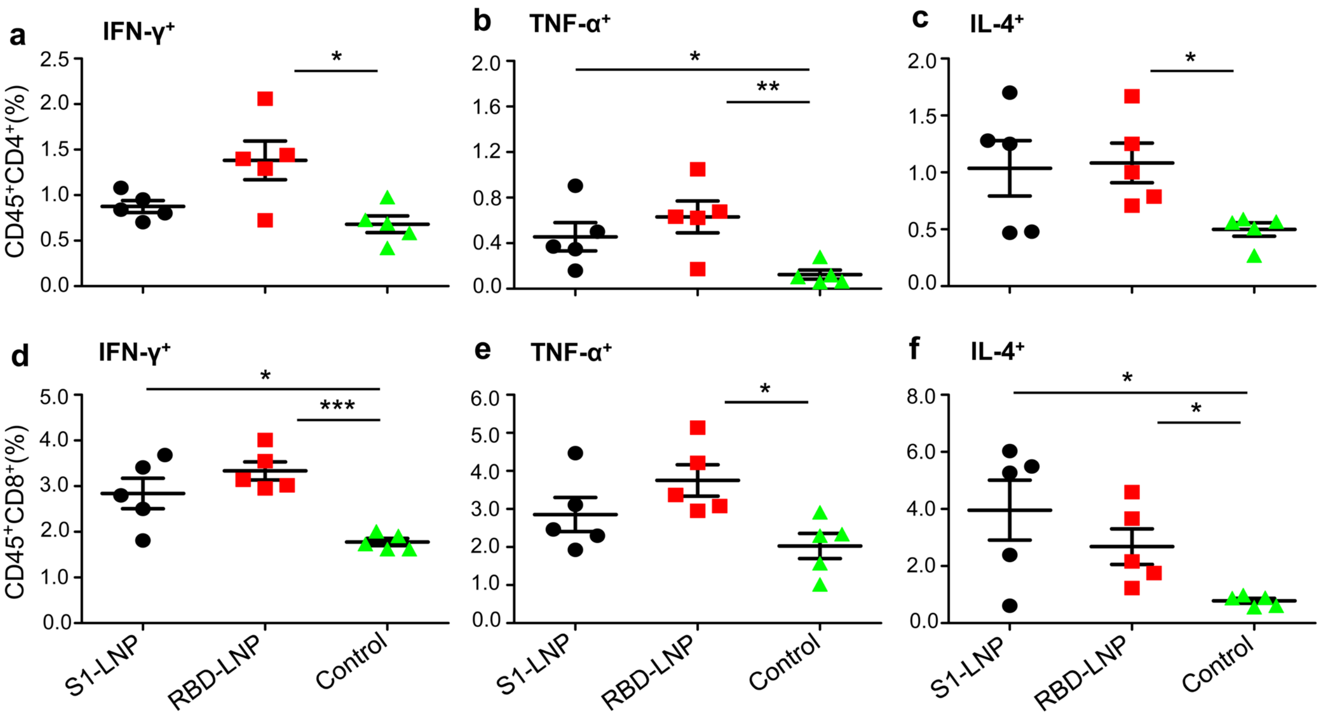

Supplementary information, Fig. S7 SARS-CoV-2 RBD mRNA-LNP elicited SARS-CoV-2 RBD-specific cellular immune responses.

a-f Mice were I.D. injected with LNP-encapsulated SARS-CoV-2 S1 or RBD mRNA (S1-LNP or RBD-LNP) (30 μg/mouse), or empty LNP (control), I.D. boosted at 4 weeks, and spleens were collected 10 days post-boost vaccination for analysis of SARS-CoV-2 RBD-specific $CD4^+$ (**a-c**) and $CD8^+$ (**d-f**) T cells by flow cytometry. To determine their frequencies, IFN-γ-, TNF-α- and IL-4-producing $CD45^+CD4^+$ T cells (**a-c**) and IFN-γ-, TNF-α- and IL-4-producing $CD45^+CD8^+$ T cells (**d-f**) were stained for the corresponding cell surface marker, and intracellular cytokines. Splenocytes were incubated with a mixture of overlapping SARS-CoV-2 RBD peptides (5 μg/ml). Significant differences (*, $P < 0.05$; **, $P < 0.01$; ***, $P < 0.001$) among different groups are shown, and the data are presented as mean ± s.e.m. of mice (n = 5). Experiments were repeated twice with similar results.

Supplementary information, Fig. S8

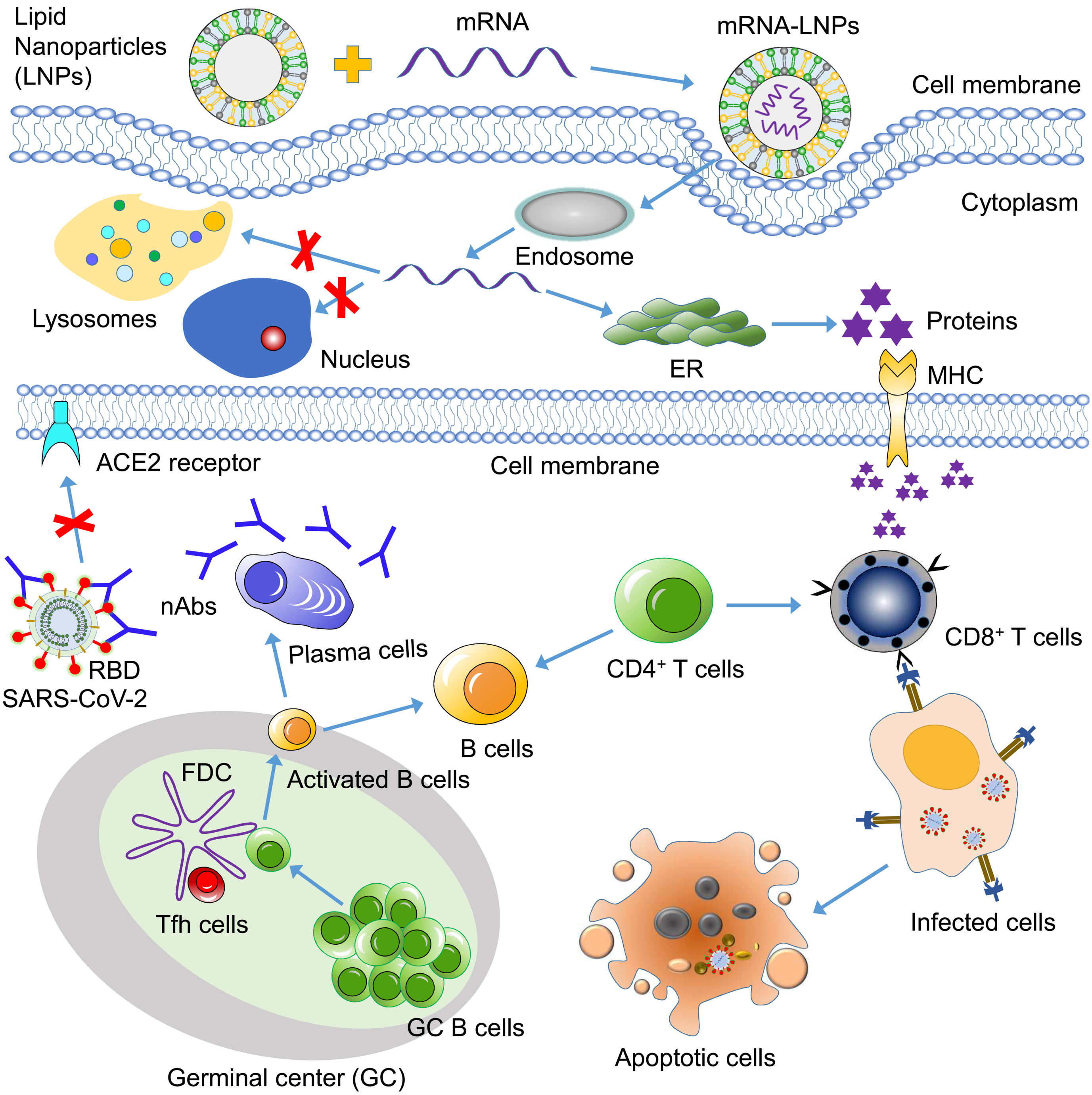

Supplementary information, Fig. S8 Proposed model of mRNA-LNP vaccine delivery and immune response induction.

SARS-CoV-2 S1 or RBD mRNA with nucleoside modification is encapsulated in LNPs to form mRNA-LNPs for delivery into individuals. The LNP-encapsulated mRNAs pass through cell membrane and cross endosomal membrane into endosome, but not nuclear membrane. The mRNA-LNPs in endosome are released into cytoplasm and the mRNAs are translated on organelles such as endoplasmic reticulum (ER) into target proteins. The mRNAs have sufficient stability against lysis by lysosomes. Both S1-mRNA and RBD mRNA can induce RBD-specific $CD4^+$ T cells to help in the production of specific antibodies and $CD8^+$ T cells to kill target cells. Tfh, GC B, and plasma cells are activated to promote or produce SARS-CoV-2-specific neutralizing antibodies (nAbs) able to neutralize SARS-CoV-2 infection and block binding of SARS-CoV-2 RBD to ACE2 receptor.

Supplementary information, Table S1 SARS-CoV-2 RBD overlapping peptides used in the study.

Number	Sequence	Number	Sequence
1	NITNLCPFGEVFNATRFASV	2	VFNATRFASVYAWNRKRISN
3	YAWNRKRISNCVADYSVLYN	4	CVADYSVLYNSASFSTFKCY
5	SASFSTFKCYGVSPTKLNDL	6	GVSPTKLNDLCFTNVYADSF
7	CFTNVYADSFVIRGDEVRQI	8	VIRGDEVRQIAPGQTGKIAD
9	APGQTGKIADYNYKLPDDFT	10	YNYKLPDDFTGCVIAWNSNN
11	GCVIAWNSNNLDSKVGGNYN	12	LDSKVGGNYNYLYRLFRKSN
13	YLYRLFRKSNLKPFERDIST	14	LKPFERDISTEIYQAGSTPC
15	EIYQAGSTPCNGVEGFNCYF	16	NGVEGFNCYFPLQSYGFQPT
17	PLQSYGFQPTNGVGYQPYRV	18	YQPYRVVVLSFELLHAPATV
